# Cognitive improvement and prefrontal network interactions in individuals with remitted bipolar disorder after transcranial infrared laser stimulation

**DOI:** 10.3389/fpsyt.2025.1547230

**Published:** 2025-01-30

**Authors:** Douglas W. Barrett, Roger E. Davis, Jennifer E. Siegel-Ramsay, Amy Bichlmeier, Jorge R. C. Almeida, F. Gonzalez-Lima

**Affiliations:** ^1^ Departments of Psychology, Psychiatry and Behavioral Sciences, and Institute for Neuroscience, The University of Texas at Austin, Austin, TX, United States; ^2^ Bipolar Disorder Center, Department of Psychiatry and Behavioral Sciences, Dell Medical School, Austin, TX, United States

**Keywords:** bipolar disorder, transcranial infrared laser stimulation, photobiomodulation, cognitive enhancement, prefrontal cortex stimulation, brain oxygenation

## Abstract

**Background:**

Converging evidence suggests that bipolar disorder (BD) involves mitochondrial dysfunction and prefrontal cortex (PFC) hypometabolism associated with cognitive impairment, which persists in remitted BD individuals. Transcranial infrared laser stimulation (TILS) provides safe, non-invasive brain stimulation that enhances PFC metabolism via photobiomodulation of mitochondrial respiration and tissue oxygenation. We tested the hypothesis that the neurocognitive deficits found in BD may be ameliorated by TILS treatments.

**Methods:**

This is the first study to explore neurocognitive effects of repeated TILS administration in BD. Using an open-label design, 29 individuals with remitted BD received six weekly TILS treatments. Working memory and attention were assessed with trail-making and 2-back tasks sensitive to TILS cognitive effects in individuals with BD. Changes in PFC network interactions were measured with functional near-infrared spectroscopy (fNIRS) because this method can measure TILS effects on oxygen metabolism in the PFC of individuals with BD.

**Results:**

Participants reported no adverse effects from treatment, confirming the safety of this intervention in individuals with BD. Cognitive test results showed that in people with remitted BD, TILS was effective at improving cognition, i.e., enhanced speed and accuracy in tasks reflecting cognitive flexibility, working memory, and attentional control. Antipsychotic medication improved TILS cognitive effects. The fNIRS results showed a significant reduction in PFC network correlations of oxygenated hemoglobin changes driven by cognitive task performance. The right-hemisphere frontopolar cortex showed greater TILS effects than its left-hemisphere counterpart.

**Conclusions:**

Repeated TILS is a safe intervention to improve cognition in people with remitted BD. Continued antipsychotic medication may have contributed to the cognitive improvement. To confirm TILS efficacy, a sham-controlled, double-blinded randomized trial is needed.

**Clinical trial registration:**

https://clinicaltrials.gov/, identifier NCT05354895.

## Introduction

Converging evidence suggests, at least in part, that bipolar disorder (BD) involves mitochondrial dysfunction (1) and reduced energy metabolism in prefrontal cortex (PFC) (2). Disruptions in brain networks mediating cognition and emotion (3) may underlie the deficits found in BD. These deficits are found even in individuals with remitted, non-cycling (euthymic) BD (4). Current clinical interventions for BD are mainly targeted for mood stabilization instead of treatment of mitochondrial deficits, and a small number of studies have focused on cognitive impairment (5).

Different neuromodulation strategies have been recently introduced in BD research (6). Transcranial infrared laser stimulation (TILS) is a safe, non-invasive low-level laser therapy that enhances brain metabolic energy production by delivering photons at 1064 nm wavelength through the forehead to the PFC [for review, see Gonzalez-Lima (7)]. The way TILS works differs from magnetic and electrical brain stimulation, such as transcranial magnetic stimulation or electroconvulsive therapy. While magnetic and electrical stimulation alter the electrical excitability of cell membranes, TILS works by having photons directly absorbed by mitochondria, which increases oxygen consumption and improves blood flow to enhance cognitive brain function. We chose TILS for BD patients because of its mitochondrial mechanism of action, which could help reduce certain cognitive impairments associated with BD by enhancing mitochondrial energy production in the PFC.

A form of transcranial photobiomodulation, TILS targets the mitochondrial enzyme cytochrome-c-oxidase (CCO), causing photo-oxidation of CCO and increasing tissue oxygenation in the PFC (8). TILS has been estimated to penetrate the PFC gray matter via a tissue optical window to infrared light at 1064 nm (9). We have previously demonstrated beneficial cognitive and emotional effects resulting from TILS (10–13), as well as beneficial effects on brain mitochondrial and oxygen metabolism (13–16). We recently extended these findings of improvements in cognition and PFC energy metabolism to older euthymic adults with BD (17, 18). TILS may alleviate some of the cognitive deficits found in BD by facilitating mitochondrial energy production and stabilizing dysfunctional neural networks in the PFC. We have previously studied the cognitive effects of single and repeated TILS administrations for five weekly treatments in older participants with and without BD (17, 19). The effects of TILS were stronger after repeated weekly administrations. For this reason, we chose to examine the effects of repeated administrations in this study.

Our hypothesis was that the neurocognitive deficits found in BD may be ameliorated by weekly TILS treatments. Working memory and attention were assessed with the trail-making task and 2-back task, because these tasks appear sensitive to TILS cognitive effects in individuals with BD (17). Changes in PFC network interactions were measured with functional near-infrared spectroscopy (fNIRS) because NIRS-based optical methods are non-invasive and capable of measuring TILS effects on oxygen metabolism in the PFC of individuals with BD and healthy controls (13, 18). This was an open-label study in which all participants received TILS treatment. It is the first study to explore the effects of repeated TILS on fNIRS network measures in individuals with remitted BD.

## Methods and materials

### Participants

All procedures were approved by the University of Texas at Austin (UT Austin) Institutional Review Board and complied with National Institute of Health (NIH) and Declaration of Helsinki guidelines on human research.

**Figure 1:**
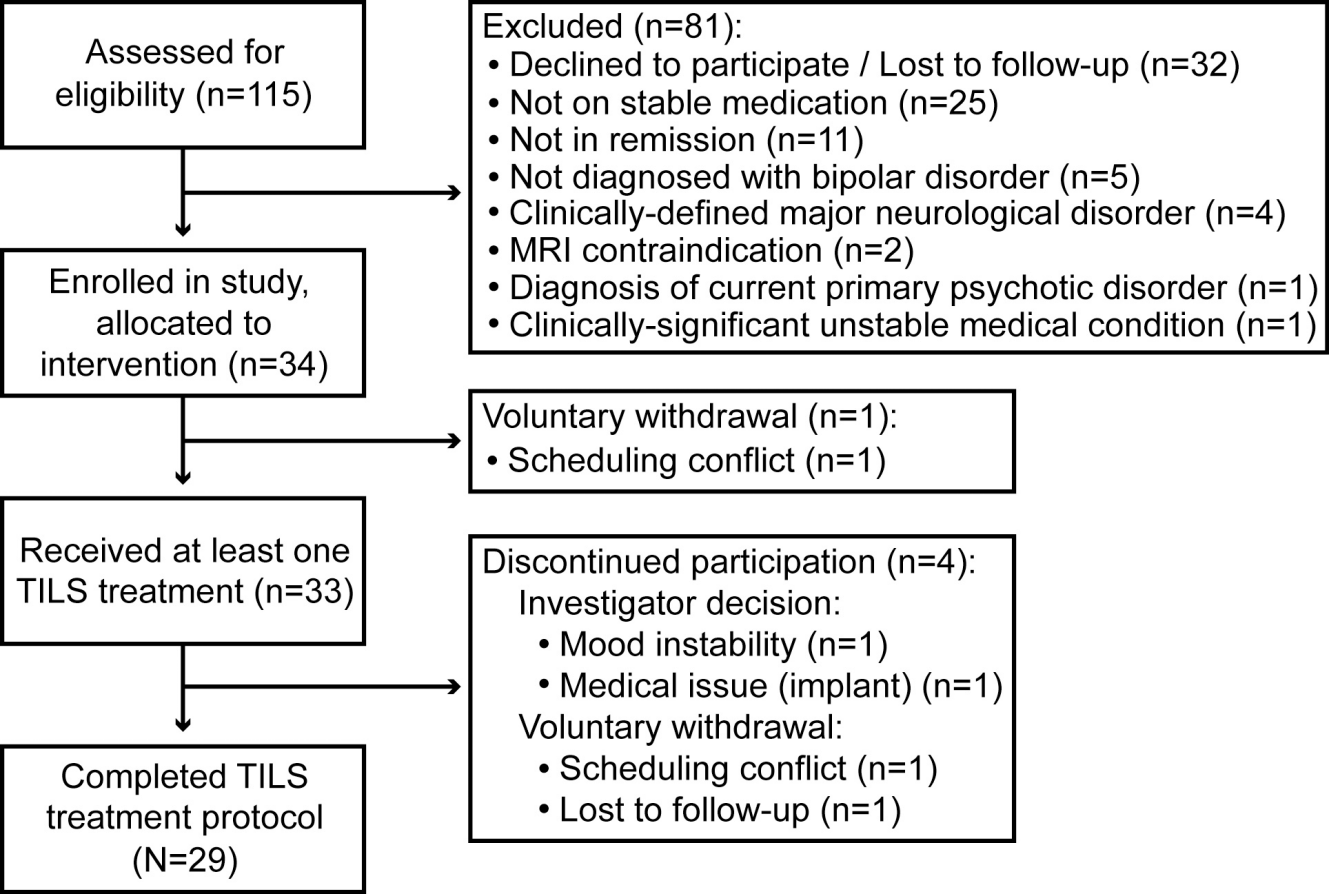
CONSORT diagram. Flow of participants through each stage of the experiment is shown.

Participants were N=29 remitted participants with BD (type I: n=22; type II: n=7), recruited through the Bipolar Disorder Center at Dell Medical School in Austin. The participants comprised n=18 females and n=11 males with an average age of 36 years (range: 19-70 years old). Participants were considered for inclusion if they were: (1) Age 18 years or above; (2) Diagnosed with either Type I or II bipolar disorder; (3) Taking at least one FDA-approved mood stabilizer for at least six weeks; (4) In remission (mood-stabilized, i.e. non-cycling), with a score on the Montgomery-Åsberg Depression Rating Scale (MADRS) ≤ 12 and a score on the Young Mania Rating Scale (YMRS) ≤ 7. Participants who changed or stopped medication partway through the study or showed signs of mood instability were discontinued. A detailed list of all inclusion and exclusion criteria for the study is shown in **Supplementary Table 1**. **Figure 1** shows a CONSORT diagram describing participant enrollment.

### Experimental procedure

**Figure 2:**
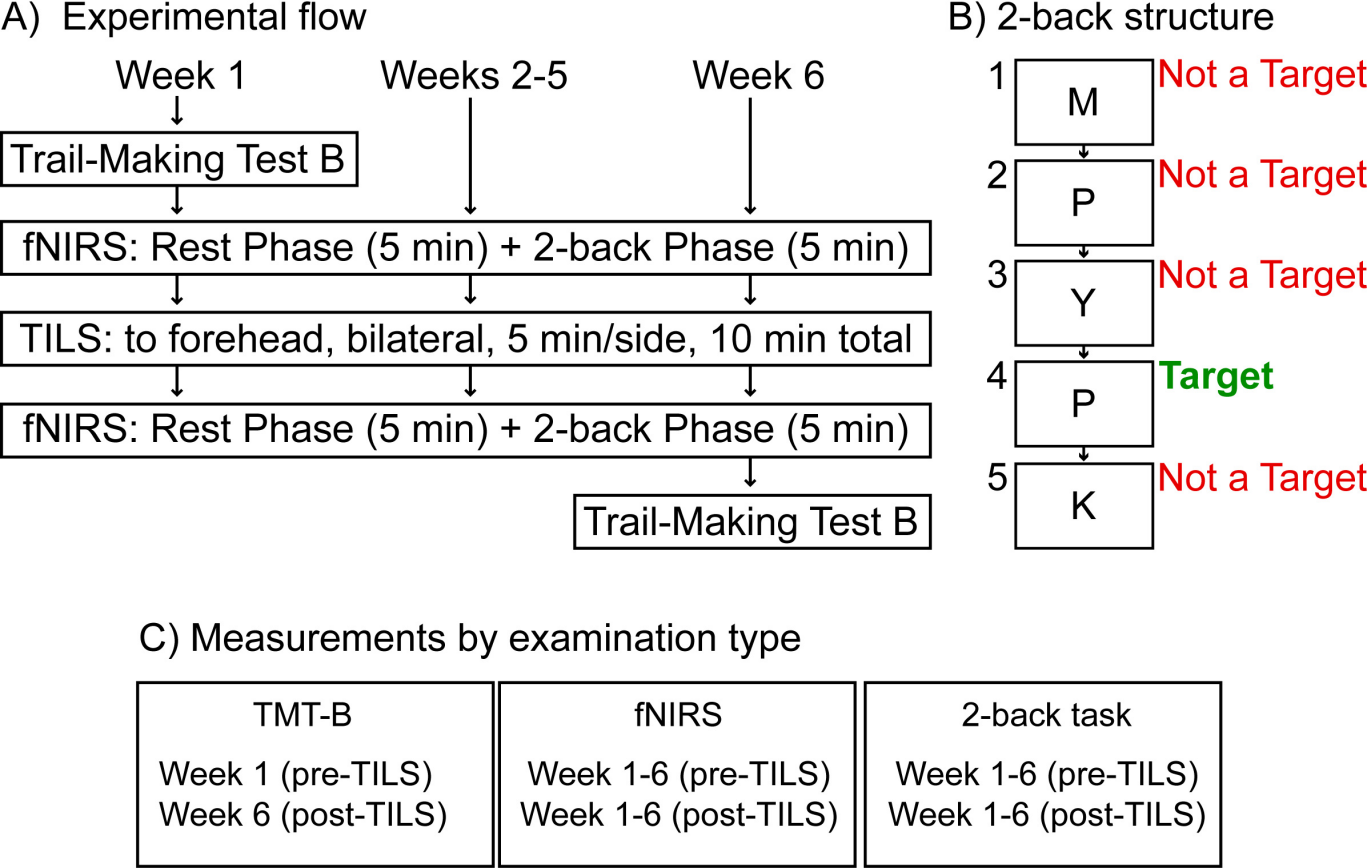
Experimental design. **(A)** Weekly TILS administration and assessments. **(B)** Structure of the 2-back cognitive assessment, showing an example of a possible sequence of letters presented onscreen (M, P, Y, P, K) during this task. **(C)** Measurements by examination type; functional near-infrared spectroscopy (fNIRS); Transcranial infrared laser stimulation (TILS).

Potential participants who indicated interest in the study were screened for inclusion and exclusion criteria by the psychiatry staff at the Bipolar Disorder Center at Dell Medical School at The University of Texas at Austin, who then obtained informed consent and enrolled the participants. All procedures and assessments reported here were conducted at the Gonzalez-Lima Lab at UT Austin. Participants also completed a series of cognitive assessments and MRI scans. The results of these measures will be reported in future manuscripts. **Figure 2A** summarizes the experimental design.

Participants received six weeks of TILS treatments, once per week. This weekly TILS treatment was chosen because it has been shown previously to improve cognitive function and modify BOLD-fMRI response in the PFC of cognitively-compromised individuals (19). The TILS device used was FDA-cleared as safe for use in humans, and it was operated in accordance with a standard operating procedure that was approved by the UT Austin Laser Safety Office. All laser operators completed a laser safety class through the Environmental Health and Safety office at UT Austin. All participants and researchers wore protective eyewear throughout each TILS session.

**Figure 3:**
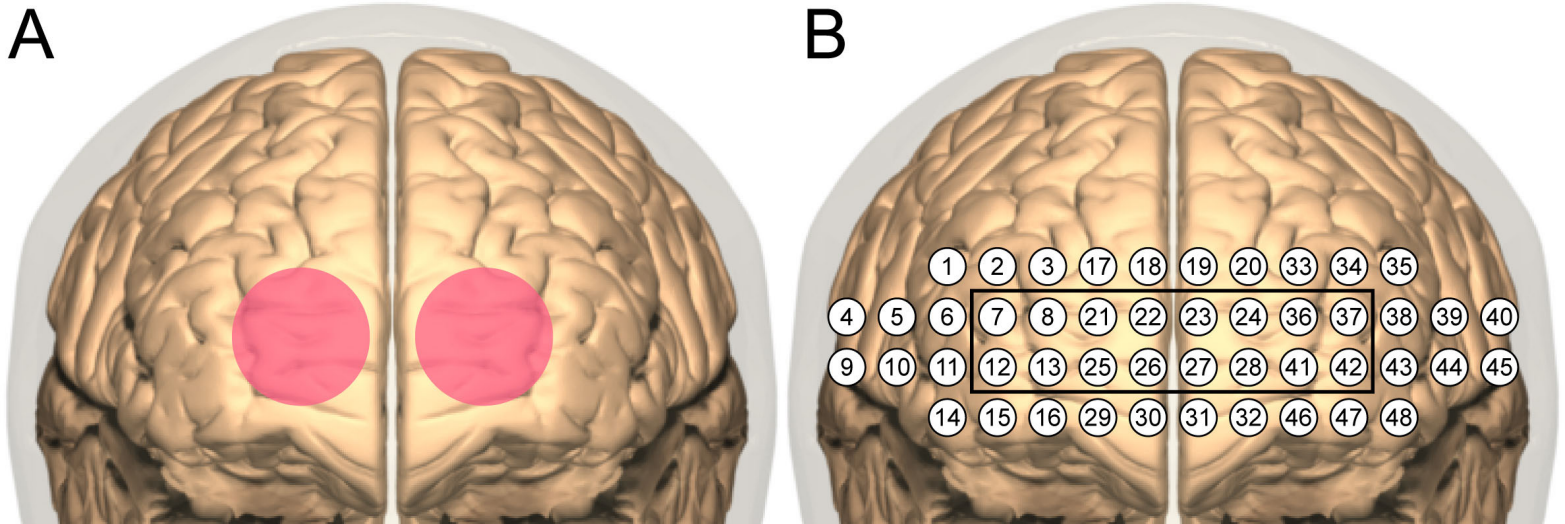
TILS and fNIRS sites. **(A)** Bilateral stimulation sites (4 cm diameter each) centered on the targeted frontopolar region of PFC. **(B)** Layout of channels in NIRSIT device. The black square indicates channels sampling the frontopolar region directly stimulated with TILS.

TILS administration consisted of the application of infrared light using a well-collimated, flat-top, continuous-wave laser (HD Laser, Cell Gen Therapeutics, Dallas, TX). The study used the same wavelength (1064 nm), irradiance (250 mW/cm^2^), cross-sectional beam area (13.6 cm^2^), total duration (10 minutes), and targeted stimulation sites as in our previous TILS studies with older bipolar adults (17, 18). The target sites were the right and left anterior PFC (frontopolar cortex, Brodmann area 10), with the laser beam centered at Fp1 and Fp2 points corresponding to the standard frontopolar EEG electrode placements. We selected these TILS parameters and points Fp1 and Fp2 as sites of stimulation because these parameters and forehead sites are the same that we have used in previous studies that have shown both cognitive and brain metabolic effects of TILS. Each site received five minutes of TILS, alternating each minute between the two sides (see **Figure 3A**). The fluence dose (or energy density) was 75 J/cm^2^ (0.25 W/cm^2^ x 300 sec) per site on the forehead, with a fluence dose of 150 J/cm^2^ across the entire forehead. This specific bilateral TILS protocol was selected because it has been previously shown to increase mitochondrial CCO and oxygenated hemoglobin in the PFC of bipolar individuals in a sham-controlled study (18). The present investigation was an open-label study in which all participants received TILS treatment; there was no sham control.

Immediately before the first TILS treatment, participants completed the Trail-Making Test part B (TMT-B). The TMT-B is a “connect-the-dots” task used to assess frontal lobe functions (20) including visual attention and cognitive flexibility. The participant is instructed to connect a series of targets as quickly as possible while still being accurate, alternating between sequential letters and numbers (1, A, 2, B, etc.). Participants completed the TMT-B again immediately after the last TILS treatment. The TMT-B was chosen because performance on this task has been previously shown to improve after TILS in bipolar individuals (17).

**Figure 4:**
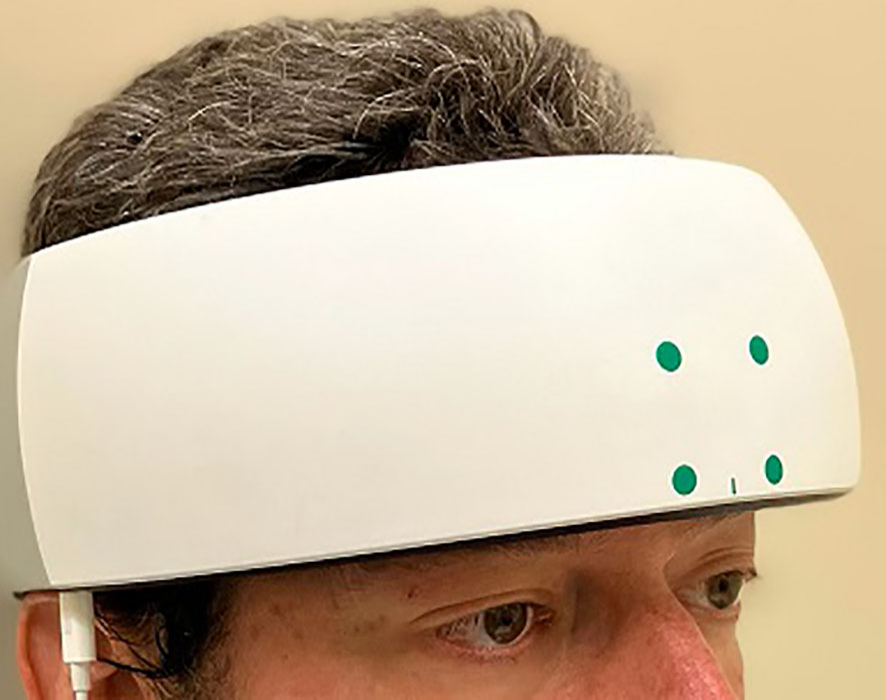
fNIRS apparatus. The NIRSIT near-infrared spectroscopy device (shown on the head of a researcher) was positioned on the forehead, centered on the midsagittal line, and secured with a Velcro strap.

Before and after each administration of TILS, network interactions in the PFC were assessed using functional near-infrared spectroscopy (fNIRS). A head-mounted device (NIRSIT: Soterix Medical Inc., Woodbridge, NJ and OBELAB Inc., Seoul, Korea) was used to measure changes in oxygenated hemoglobin concentration across the PFC. This device uses an array of light emitters and photodetectors to spectroscopically sample from 48 channels (see **Figure 3B**) with a separation of 3 cm between each pair of emitters/detectors that comprised a channel. The device was fixed firmly on the forehead and secured there using a Velcro strap (see **Figure 4**). Participants confirmed verbally that the headset was securely fastened on the head without being painful. An fNIRS method was used because it has been previously shown to detect TILS-induced changes in oxygenated hemoglobin within the PFC of healthy controls (13). We have previously demonstrated that the increases in concentration of both oxygenated hemoglobin and cytochrome-c-oxidase after TILS are highly correlated (15).

Each fNIRS session lasted approximately 10 minutes and consisted of two phases. During the first five minutes (“Rest Phase”), participants rested quietly. During the last five minutes (“2-back Phase”), participants engaged in the 2-back task, which is a commonly-used measure of working memory and executive control (21) mediated by PFC function (22). Participants were instructed to watch a series of letters presented onscreen, remember which letter was presented two trials back, and only respond if the target letter currently onscreen matched the letter from two trials back. **Figure 2B** describes the structure of the 2-back task. **Figure 2C** summarizes each measurement by examination type.

### fNIRS data processing

Spectroscopic data gathered with the NIRSIT device was processed using the NIRSIT Analysis Tool provided by OBELAB Inc. The data was processed using standard procedures reported in previous spectroscopic studies of TILS effects in the PFC (13, 15, 16, 18). Briefly, the data was subjected to a band-pass filter with limits of 0.005 Hz to 0.1 Hz to eliminate noise from sources unrelated to the signal from PFC, such as cardiovascular artifacts and respiration. Markers inserted during data acquisition were used to define two epochs for each fNIRS session: the Rest Phase (5 minutes) and the 2-back Phase (5 minutes). Average light intensity from the first five seconds of spectroscopic measurements was defined as the baseline intensity (I_0_) in the modified Beer-Lambert law, which is a mathematical relationship used to convert changes in light intensity to changes in oxygenated hemoglobin concentration and is commonly used in near-infrared spectroscopy (23). Any channel with a signal-to-noise ratio below 25 was rejected before oxygenated hemoglobin concentration was calculated. For rejection padding, any missing channel was replaced by a backup channel (an alternate pair of emitters/detectors) where possible; otherwise, the brain regional mean was used.

While it is possible to calculate changes in concentration of oxygenated hemoglobin within a single session using fNIRS, it is not possible to calculate absolute concentration, because the initial intensity measurement I_0_ in the modified Beer-Lambert law is only valid within a given session, and subsequent measurements are specifically dependent on that initial value (13). Therefore, to assess between-session (i.e. weekly) changes, an index of network interactivity was calculated using the within-session concentration changes of oxygenated hemoglobin across all 48 channels. The NIRSIT Analysis Tool software used these concentration changes to generate a 48x48 matrix of pairwise correlation coefficients, representing each pairwise combination of each of the 48 channels sampled by the NIRSIT device. Correlation matrices for the Rest Phase and 2-back Phase were computed separately. Pairwise correlation matrices were used because they are a standard method for analyzing neural network interactions in both animal and human studies (24–26).

To generate a single index of network interactivity for each subject/session/phase, the Fisher Z-transformation was used to convert each 48x48 correlation matrix to a matrix of z-scores. These z-scores were then averaged together, and the subsequent value converted back to a correlation coefficient, which represented the average overall (“all-channels”) network interactivity across the PFC, including every pairwise combination of each channel in the 48-channel array. In addition to this “overall” index of PFC network interactivity, another “stimulation site” index was calculated which focused on the frontopolar stimulation sites in the left and right hemisphere. The same Fisher Z-transformation process was used to obtain this average value for each subject but limited to only those channels defined as “frontopolar” (as seen in **Figure 2B**) in the NIRSIT Analysis Tool software. Separate values were calculated for right and left frontopolar regions, to assess whether hemispheric laterality played a role in changes in network interactivity due to TILS.

One subject/session was missing from the fNIRS dataset due to an equipment problem. This missing value was replaced using a standard mean imputation approach: data from Week 1 and Week 3 for this individual were averaged together to generate the missing data from Week 2. Corresponding phases were used to impute the missing data: e.g., Week 1, Pre-TILS, Rest Phase was averaged with Week 3, Pre-TILS, Rest Phase to generate Week 2, Pre-TILS, Rest Phase for this subject. One subject had missing 2-back data for Weeks 1 and 2 due to misunderstanding the instructions and pressing the wrong key in response to correct trials. These data were also replaced with a mean imputation, using averaged data from the corresponding time points from all other subjects. These participants received the same number of TILS treatments and cognitive assessments as the other participants, and thus they were treated identically as the rest of the participants in terms of the experimental procedure.

### Statistical analysis

The dependent variables of interest for the cognitive assessments (TMT-B and 2-back) comprised one score reflecting accuracy and another representing speed. For the TMT-B, the dependent variables were number of errors and completion time (seconds). The TMT-B dependent variables were consolidated into a rate correct score, which incorporates both accuracy and completion speed into a single metric (13, 27).

For the 2-back task, the dependent variables were the total number of correct responses (or Hits) and the response time on correct trials (in milliseconds). The dependent variables for the fNIRS data were the “all-channels” average correlation coefficient, reflecting overall network interactions in the PFC, and the frontopolar average correlation coefficients (right and left), representing stimulation site network interactions within the TILS stimulation sites.

The TMT-B data was analyzed using paired t-tests comparing speed and accuracy mean scores at Pre-TILS (Week 1) vs. Post-TILS (Week 6). The 2-back data was analyzed during each week using repeated measures ANOVA with two within-subject variables: PrePost (2 levels: Pre-TILS vs. Post-TILS) and Weeks (6 levels: weeks 1, 2, 3, 4, 5, 6). The fNIRS all-channels network interactivity data was analyzed using repeated measures ANOVA with three within-subject variables: Phase (2 levels: Rest vs. 2-back), PrePost (2 levels: Pre-TILS vs. Post-TILS) and Weeks (6 levels: weeks 1, 2, 3, 4, 5, 6). The fNIRS frontopolar network interactivity data was also analyzed with repeated measures ANOVA but included an additional within-subject variable: Laterality (2 levels: left vs. right).

To test whether participant medication usage had an effect on treatment response to TILS, a series of repeated measures ANOVAs with one within-subject variable [2 levels: Pre-TILS (Week 1) vs. Post-TILS (Week 6)] and one between-subject variable (medication status) was performed on the rate correct score dependent variable. Separate repeated measures ANOVAs were performed using four between-subject variables, corresponding to whether or not a participant was medicated with antipsychotics, antidepressants, ADHD medications, or benzodiazepines (13, 27).

There was no between-subject treatment group variable for these analyses, since all subjects received TILS treatment. The free, open-source statistical package jamovi (28) was used to perform the statistical analyses. The assumption of normality of the TMT-B data in the paired t-tests was checked using the Shapiro-Wilk test (29). The assumption of sphericity of the 2-back and fNIRS data in the repeated measures ANOVAs was checked using Mauchly’s W (30). The Greenhouse-Geisser correction for non-spherical data (31) was used where applicable. A finding was considered statistically significant for p-values below 0.05. Estimates of effect size were calculated as Cohen’s d and partial eta squared (32) for paired t-tests and repeated measures ANOVAs, respectively. For Cohen’s d, a value of 0.2 indicates a small effect size, 0.5 a medium effect size, and 0.8 a large effect size; for partial eta squared, a value of 0.01 indicates a small effect size, 0.06 a medium effect size, and 0.14 a large effect size (33).

## Results

Demographic details, the psychiatric comorbidities, and psychotropic medication usage for participants are shown in **Table 1**. **Supplementary Table 2** lists all medications for all participants.

**Table 1:** Demographic data of participants, psychiatric comorbidities, and psychotropic medication use.

Demographics	N (%)
Total participants	29 (100)
Age (mean ± SD, years)	35.9 ± 13.6
Sex	
Female	18 (62.1%)
Male	11 (37.9%)
Race	
White	20 (69.0%)
Asian	3 (10.3%)
Other*	4 (13.8%)
Black/African American	1 (3.4%)
Multi-racial**	1 (3.4%)
Native Hawaiian/Other Pacific Islander	0
American Indian/Alaska Native	0
Ethnicity	
Hispanic	4 (13.8%)
Non-Hispanic	25 (86.2%)
Education Level (Highest Attained)	
High school diploma or GED	0
At least one year of college, no degree	7 (24.1%)
Associate’s Degree	2 (6.9%)
Bachelor’s Degree	10 (34.5%)
Graduate School, no degree	2 (6.9%)
Master’s Degree	5 (17.2%)
Doctoral Degree	1 (3.4%)
Professional Degree	1 (3.4%)
Not Reported	1 (3.4%)
Bipolar Disorder Type	
Bipolar disorder type I	21 (72.4%)
Bipolar disorder type II	8 (27.6%)
Mood Assessment at Baseline (pre-tPBM)	
Montgomery-Åsberg Depression Rating Scale (MADRS)	2.62 ± 2.92
Young Mania Rating Scale (YMRS)	0.72 ± 1.00
Psychiatric Comorbidity	
Cannabis Use Disorder	4 (13.8%)
Generalized Anxiety Disorder	3 (10.3%)
Social Anxiety Disorder	3 (10.3%)
Specific Phobia	3 (10.3%)
Attention Deficit/Hyperactivity Disorder (ADHD)	2 (6.9%)
Premenstrual Dysphoric Disorder (PMDD)	2 (6.9%)
Post-Traumatic Stress Disorder (PTSD)	1 (3.4%)
Psychotropic Medications	
Mood Stabilizers***	29 (100%)
Antipsychotics	13 (44.8%)
Antidepressants	10 (34.8%)
ADHD Medications	6 (20.7%)
Benzodiazepines	5 (17.2%)

*Other: This category includes one participant who identified as Berber, one as Persian, and two participants who did not provide a description.

**Multi-racial: One participant identified as both White and Asian.

***Mood Stabilizers: This category includes lithium and/or lamotrigine taken by study participants.

### Trail-making test performance improvement

**Figure 5:**
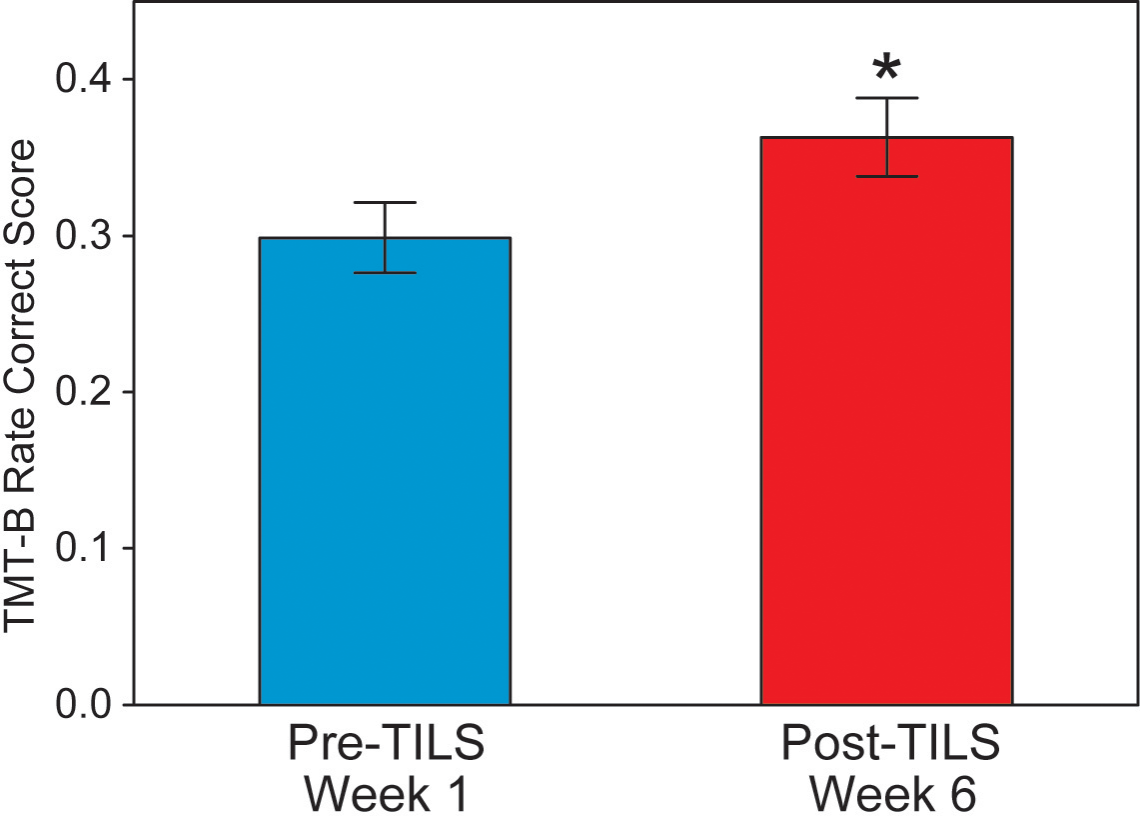
Trail-making test part B results. Rate correct score, before and after 6 weeks of TILS treatment (mean +/- SE). Paired t-test, *p<0.01.

A paired t-test on the rate correct scores on the TMT-B showed a significant effect, t(28)=3.65, p=0.0011, Cohen’s d=0.68, indicating that the participants showed significant improvement on the task. **Figure 5** shows this improvement (an increase of 22%) from Week 1 Pre-TILS to Week 6 Post-TILS. The rate correct scores were normally distributed (Shapiro-Wilk test for normality, p>0.05).

### 2-back task performance improvement

**Figure 6:**
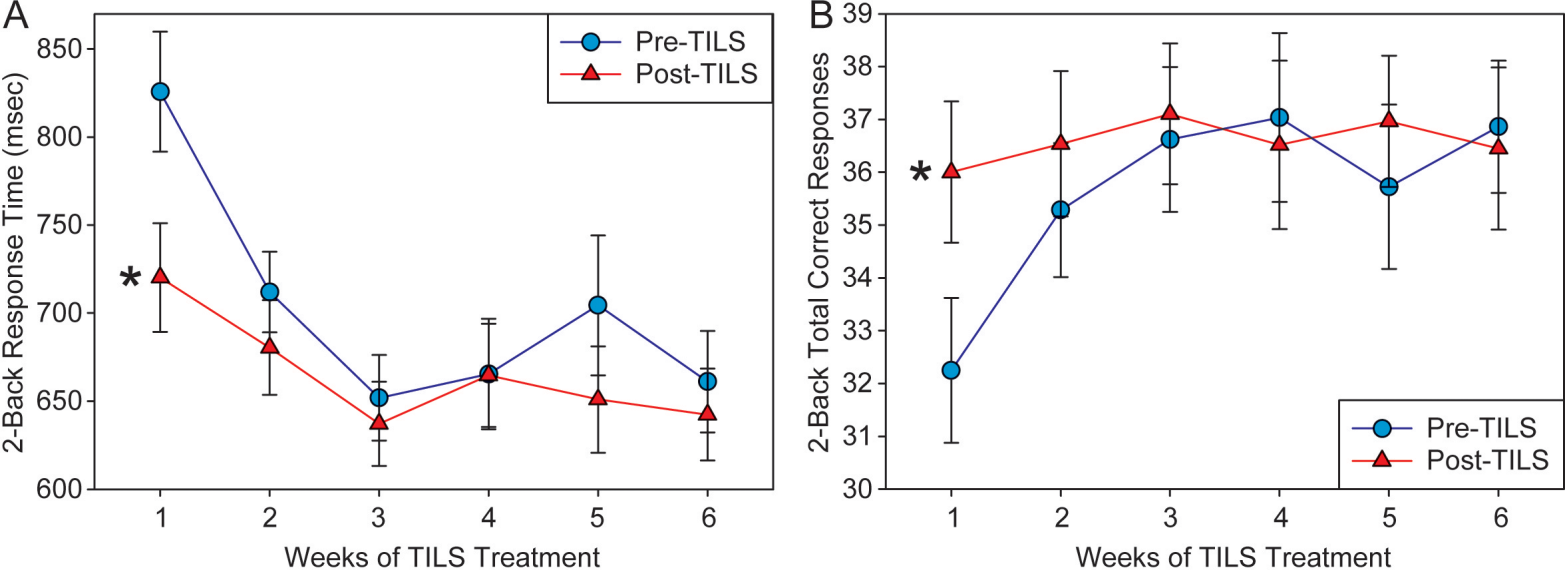
2-back task results. **(A)** Response time (in milliseconds) to correct trials in the 2-back task, pre- and post-TILS, across 6 weeks of TILS treatment (mean +/- SE). **(B)** Number of correct responses on the 2-back task, pre- and post-TILS, across 6 weeks of TILS treatment (mean +/- SE). Repeated measures ANOVA, *p<0.001.

A repeated measures ANOVA on correct trial reaction time during the 2-back task showed a significant main effect of Weeks, F(5,140)=11.32, p<0.001, partial eta squared=0.288, indicating progressively faster speed of performance; as well as a significant main effect of PrePost, F(1,28)=13.06, p=0.001, partial eta squared=0.318, indicating better performance at Post-TILS. There was also a significant interaction of Weeks by PrePost, F(5,140)=3.43, p=0.006, partial eta squared=0.109. **Figure 6A** shows the significant improvement in reaction time. From Week 1 pre-TILS to Week 6 post-TILS, there was a 22% reduction in reaction time. A test of sphericity (Mauchly’s W) found that the data was not spherical; however, applying the Greenhouse-Geiser sphericity correction resulted in no change in the pattern of significant findings (Weeks: p<0.001; PrePost: p=0.001; Weeks x PrePost: p=0.017). *Post-hoc* paired t-tests confirmed that while PrePost speed on Week 1 was significantly different (p<0.001), the data converged and was not significant on Week 2 (p=0.104).

A repeated measures ANOVA on the total number of correct responses in the 2-back test revealed a significant main effect of Weeks, F(5,140)=2.94, p=0.015, partial eta squared=0.095, as well as a significant interaction between Weeks and PrePost, F(5,140)=4.59, p<0.001, partial eta squared=0.141. The main effect of PrePost approached significance, F(1,28)=3.58, p=0.069, partial eta squared=0.113. **Figure 6B** shows the significant improvement in performance in total correct responses. From Week 1 pre-TILS to Week 6 post-TILS, there was a 13% increase in total correct trials. A test of sphericity (Mauchly’s W) found that the data was not spherical; however, applying the Greenhouse-Geiser sphericity correction resulted in no change in the pattern of significant findings (Weeks: p=0.030; Weeks x PrePost: p=0.003; PrePost: p=0.069). *Post-hoc* paired t-tests confirmed that while PrePost correct response on Week 1 was significantly different (p<0.001), the data converged and was not significant on Week 2 (p=0.079).

### Prefrontal overall network interactions with rest phase and 2-back task

**Figure 7:**
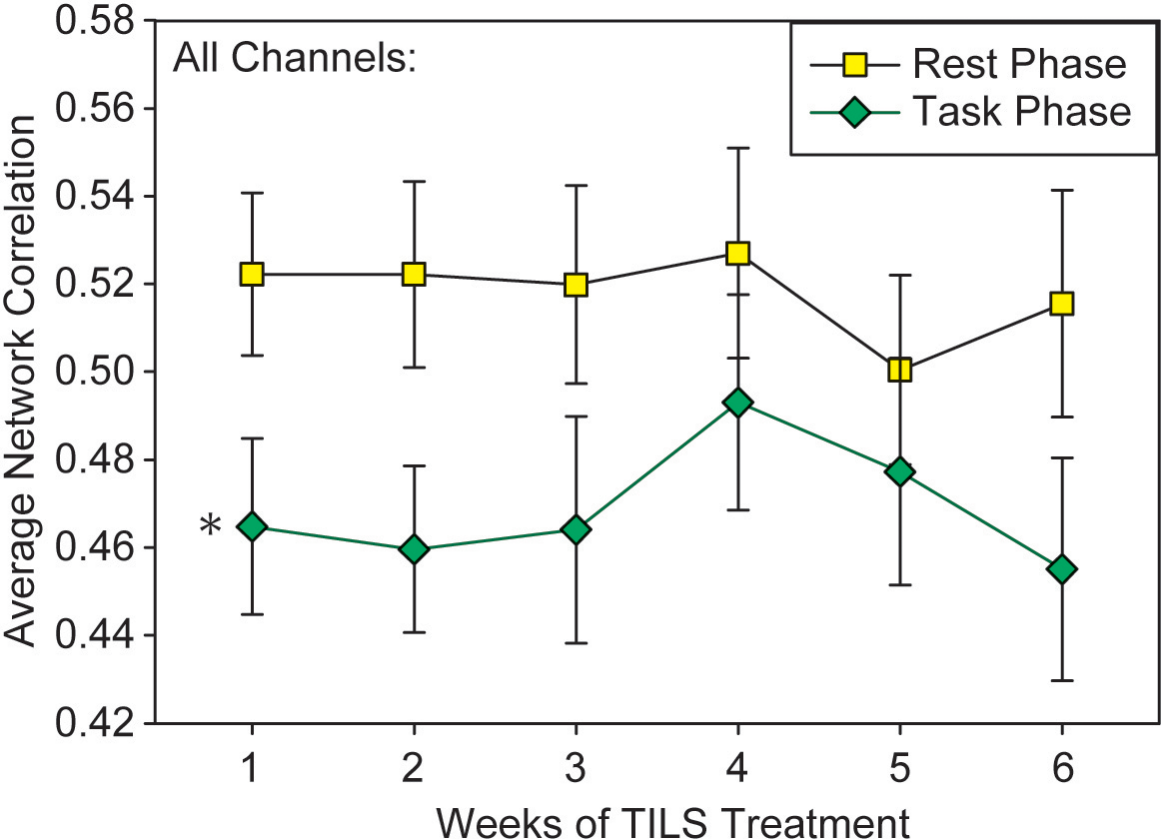
Network interactivity index results. Average pairwise correlations across all frontal regions during the 5-minute rest phase and 5-minute 2-back task (mean +/- SE). Repeated measures ANOVA, *p<0.001.

A repeated measures ANOVA on the “all-channels” index of PFC network interactivity, with the within-subject variables Weeks, PrePost, and Phase, revealed a single significant finding: a main effect of Phase, F(1,28)=14.15, p<0.001, partial eta squared=0.336. There was no significant main effect of PrePost (p=0.19) or Weeks (p=0.86), nor any significant variables interactions. **Figure 7** shows how the average network correlation across all PFC regions was significantly lower during the 2-back task phase as compared to the rest phase. A test of sphericity (Mauchly’s W) confirmed that this assumption was met for all variables in the repeated measures ANOVA. *Post-hoc* paired t-tests showed that while Phase was significantly different on Weeks 1-3 (p<0.05), the data converged and Phase was not significant on Week 4 (p=0.146).

### Frontopolar stimulation site network interactions

**Figure 8:**
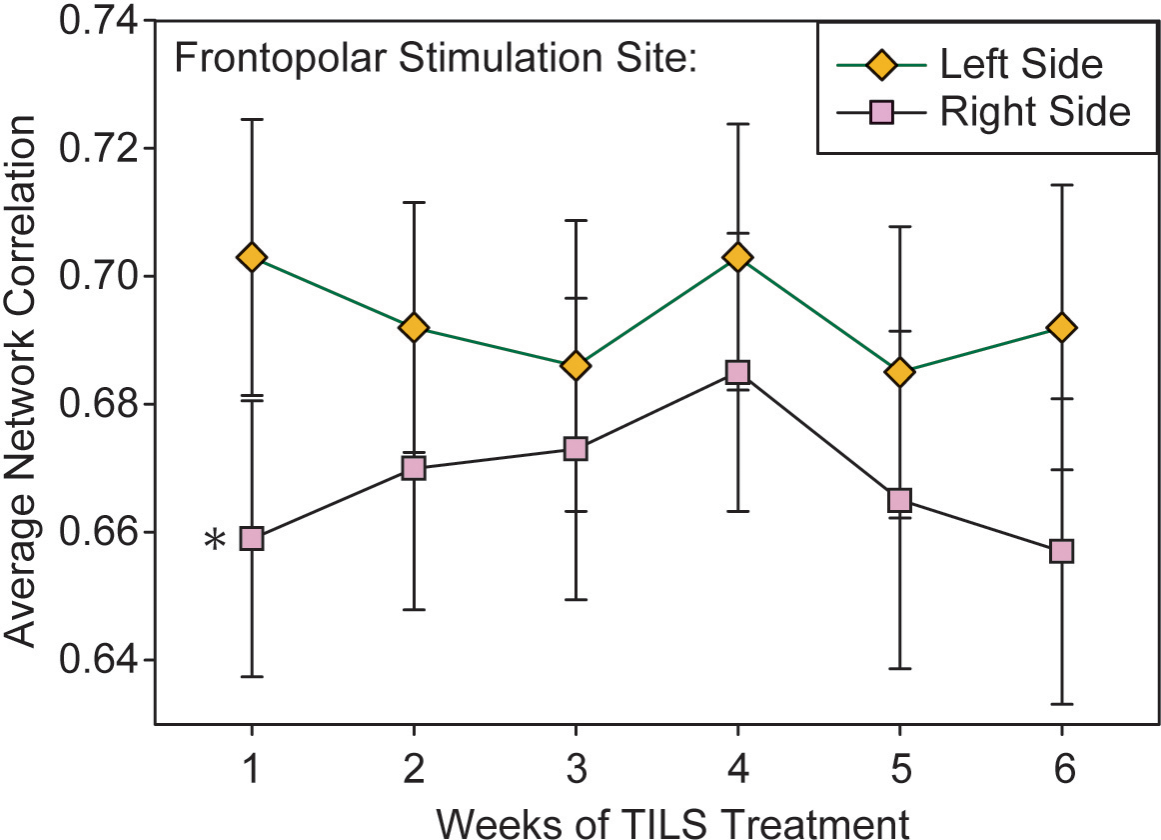
Lateralized effect in frontopolar network interactivity. Average pairwise correlations within the frontopolar region directly stimulated with TILS, as measured in left and right hemispheres. Repeated measures ANOVA, *p<0.05.

A repeated measures ANOVA on the index of local network interactivity within the frontopolar stimulation site was conducted with the within-subject variables Weeks, PrePost, Phase, and Laterality. It revealed a significant main effect of Phase, F(1,28)=7.102, p=0.013, partial eta squared=0.20, which was similar to the effect seen in the “all-channels” overall index. It also found a significant main effect of Laterality, F(1,28)=6.164, p=0.019, partial eta squared=0.18, in which the index of network interactivity was reduced in the right frontopolar region as compared to the left (**Figure 8**). The overall magnitude of the frontopolar site index (**Figure 8**) was greater than the overall PFC index (**Figure 7**). A test of sphericity (Mauchly’s W) confirmed that this assumption was met for all variables in the repeated measures ANOVA. *Post-hoc* paired t-tests showed that while Laterality was significantly different on Week 1 (p=0.037), the data converged and Laterality was not significant on Week 4 (p=0.157).

**Table 2** shows brain-behavior correlations between changes in cognitive function score (pre-post) and changes in fNIRS frontopolar connectivity. The only difference between pre-TILS and post-TILS was a hemispheric laterality observed as an opposite pattern of correlation values between rest (Phase 1) and 2-back (Phase 2) in the right hemisphere, which was not present in the left hemisphere.

**Table 2:** Brain-behavior correlations (r) between fNIRS data and TMT-B rate correct score.

fNIRS	Pre-TILS (Visit 1)	Post-TILS (Visit 6)
Phase 1, Left side	0.190	0.293
Phase 2, Left side	-0.091	0.057
Phase 1, Right side	0.253	0.069
Phase 2, Right side	0.039	0.228

### Medication status and treatment response

**Figure 9:**
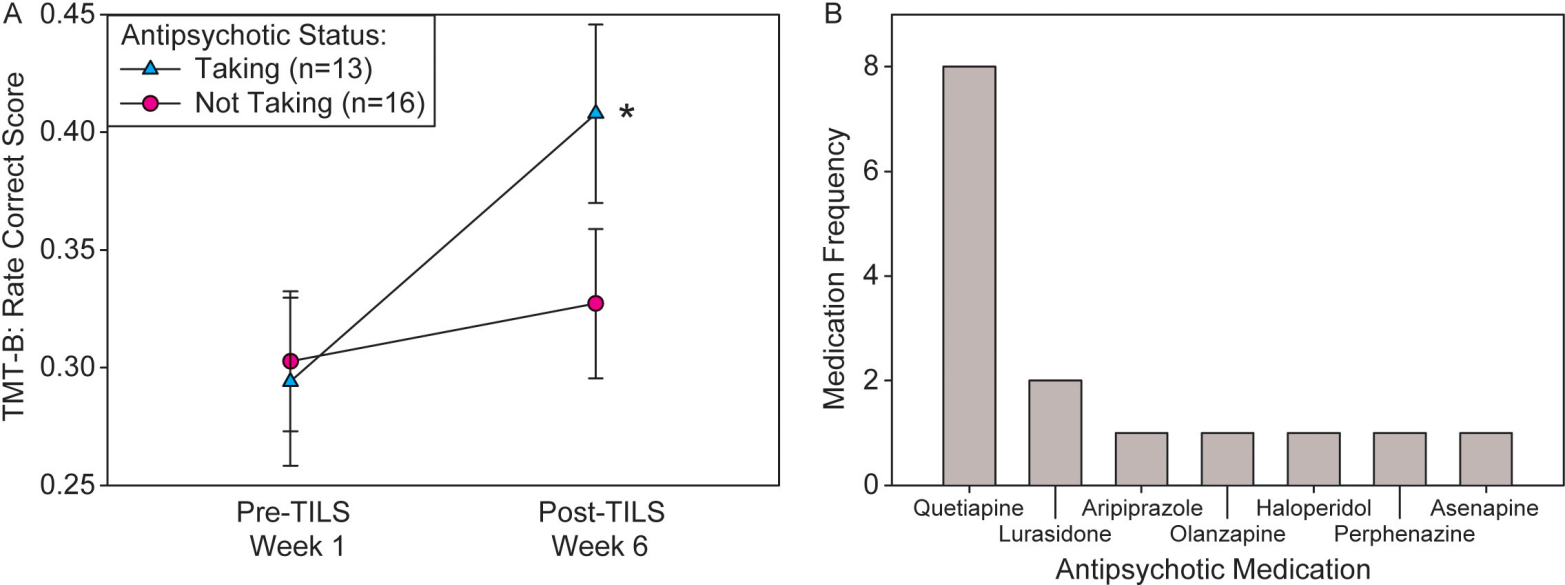
Interaction of antipsychotic medication status and treatment response. **(A)** Significant interaction of PrePost by Medication on rate correct score (mean +/- SE) in the TMT-B. Participants medicated with antipsychotics showed significantly greater cognitive improvement than unmedicated participants. Repeated measures ANOVA, *p<0.01. **(B)** Frequency distribution of each specific type of antipsychotic medication prescribed to participants.

Of the four repeated measures ANOVAs assessing whether medication status had an effect on treatment response to TILS, only antipsychotic medication status showed a significant interaction of PrePost by Medication, F(1,27)=7.90, p=0.009, partial eta squared=0.226, with medicated participants showing greater improvement in the TMT-B than unmedicated participants. **Figure 9A** shows the significant interaction in the rate correct score, and **Figure 9B** shows a frequency distribution of the exact type of antipsychotic medication prescribed to the n=13 medicated individuals. Some individuals were prescribed more than one type of antipsychotic (all medication prescribed to all participants can be found in **Supplementary Table 2**).

## Discussion

### Cognitive effects

Participants reported no adverse effects from the TILS treatment, confirming the safety of this noninvasive intervention in individuals with BD. The cognitive test results showed that in people with remitted BD, TILS was effective at improving cognition with medium to large effect sizes, i.e., enhanced speed and accuracy in tasks reflecting cognitive flexibility, working memory, and attentional control. In the 2-back task, this effect was most pronounced in the acute phase (Week 1) of the experiment, with cognitive scores leveling off in subsequent weeks. This convergence of Pre-TILS and Post-TILS scores was likely due to ceiling effects (in correct responses) and floor effects (in reaction time) in the 2-back task, as well as practice effects. Interpretation of the 2-back results is complicated by the fact that “Pre-TILS” scores in later weeks are also likely benefiting from previous weeks of treatment. Thus, each subsequent “Pre-TILS” score is not purely “Pre,” but also represents a one-week-later follow-up from the previous TILS treatment. These issues are less important for the TMT-B, because it was given only twice (before the first TILS treatment and after the last TILS treatment).

In our previous work (17), older participants with euthymic bipolar disorder showed cognitive benefits from TILS, in assessments of cognitive flexibility, working memory and sustained attention, including the TMT-B. Participants with euthymic bipolar disorder score significantly lower on the TMT-B than healthy controls (34), and in general, 83% of individuals with BD show “serious” cognitive and functional deficits, which represents the highest percentage found in all mood disorders (35). Furthermore, these cognitive deficits are found even in individuals with euthymic BD (4), and therefore, the underlying process impairing cognition in BD is not necessarily related to mood cycling.

### Mitochondrial mechanisms of TILS cognitive effects

Cognitive deficits in BD likely involve mitochondrial dysfunction. Abnormalities in mitochondrial DNA (36), mitochondrial structure (37), mitochondrial function (38), and brain energy metabolism in general (39) are well-established in BD, leading to the hypothesis that mitochondrial impairment plays a causal role in the disease pathology of BD (40). It is possible that the progressive deterioration in cognition found in older adults with euthymic BP (41) is related to the gradual accumulation of oxidative damage with age, with these mitochondrial deficits leading to a kind of accelerated aging effect (42, 43).

This line of evidence implicates mitochondrial function as a therapeutic target in BD. We have previously shown (13, 15, 16) that TILS can result in beneficial increases of oxygenated hemoglobin and oxidized cytochrome-c-oxidase, a key component of the mitochondria and the rate-limiting enzyme for energy production in the electron transport chain (44). This increase in oxidized cytochrome-c-oxidase resulting from TILS is greater in older individuals (16). Our previous sham-controlled study (18) in older adults with euthymic BD demonstrated that a single session of TILS resulted in increases in the concentration of oxygenated hemoglobin and oxidized cytochrome-c-oxidase in PFC as determined by broad-band near-infrared spectroscopy (bbNIRS). But it is not possible to use bbNIRS or fNIRS to assess across-session concentration of chromophores, because these optical methods involve recalibrating the baseline concentration value each session, which precludes calculation of absolute concentrations. Nevertheless, the totality of evidence implies that increased mitochondrial function with concomitant increased brain oxygenation is likely the underlying neurobiological mechanism by which the cognitive benefits seen in the present BD study are made manifest.

### Network effects

The significant reduction in network interactions measured by the correlation analysis of oxygenated hemoglobin changes from the resting phase to the task phase likely reflects neuroplastic changes in PFC, driven by increased cognitive demand of the 2-back task. Shifting from rest to task resulted in an overall decrease in the “all-channels” index, which is consistent with findings from BOLD-fMRI work showing that during the 2-back task, average whole-brain measures of functional connectivity decrease as compared to the resting state (45). Functionally-specialized regions like the PFC serve as discrete information processing units, making specific contributions to cognition. A shift from global to local processing in fNIRS covariance data is similar to that found in studies using electroencephalography (EEG), in which moving from the resting state to active processing of information is accompanied by a shift from synchronized to desynchronized EEG signals (46), as discrete brain regions are recruited to serve their specific functional purpose. However, TILS did not significantly change network interactivity, either acutely (PrePost) or chronically (Weeks), indicating that PFC network interactivity as measured by correlations of oxygenated hemoglobin changes may not be sensitive enough to detect TILS treatment effects or serve as a useful proxy for TILS-induced changes in brain metabolic activity.

The more localized index of network interactivity, which focused on the frontopolar stimulation sites, showed a similar significant main effect of Phase, as well as an effect of hemispheric laterality: right-hemisphere frontopolar cortex showed reduced intra-regional network interactivity than its left-hemisphere counterpart. PFC in general is marked by lateral asymmetries in anatomical connections (47), functional connectivity with other brain regions and networks (48), and functional role in cognition (49), making interpretation of this finding difficult. However, previous TILS studies have found that stimulation of the right PFC is more effective than stimulation of the left PFC at improving cognitive tasks related to attention and working memory in healthy controls and people with increased depressive symptoms, suggesting that the right PFC is more responsive to TILS cognitive effects (50, 51). The present study supports this interpretation because while both frontopolar sites received the same amount of TILS exposure, the right site showed a more pronounced cognitive-task evoked network change. In addition, individuals with BD show greater right-sided functional connectivity between amygdala and PFC in response to emotional faces, suggesting a greater contribution of the right hemisphere to emotional regulation (52, 53). Right hemisphere frontal lobe function is linked to performance on both the TMT-B (54) and the 2-back task (55), so task-related lateralization of function may have also contributed to these findings.

While it is notable that the network interactivity index within frontopolar cortex was larger than the overall PFC index, this is to be expected, as nearby spectroscopy channels tend to be more highly correlated and may sample some of the same tissue as the emitted light travels on an arc-shaped path through the cortex. These nearby channels also likely share some of the same blood vessels supplying the oxygenated hemoglobin that was used to calculate the correlation matrices. However, some of the increased interactivity seen local to the stimulation site may be due to the stimulation itself. We recently found that a single TILS treatment to the entire rat brain resulted in an increase in overall network interconnectivity across the entire brain, as measured by increased numbers of significant inter-regional correlations in cytochrome-c-oxidase enzymatic activity (56). This finding suggests that TILS can induce bioenergetic changes in infrared-stimulated brain tissue, leading to increased neuroplasticity and neuromodulation of functionally coupled neural networks.

### Medication effects

Antipsychotic medication status showed a significant interaction with treatment, indicating that it improved treatment response to TILS. The other classes of medication did not show a significant interaction. While the percentages taking antipsychotics and antidepressants were similar (45% vs. 35%), only the antipsychotics showed a significant effect. However, the other medications assessed (ADHD medications, benzodiazepines) were relatively unbalanced in their distributions (see **Table 1**), which may have affected the statistical power, resulting in negative findings. The proportion of patients taking anti-ADHD medications was higher than the rate of comorbid ADHD (**Table 1**), because these patients were prescribed anti-ADHD medications off-label by their physicians. Mood stabilizers and antipsychotics are commonly used to treat bipolar disorder and may also help enhance cognitive function. As all participants were on mood stabilizers, no differences would be expected in this group. However, significant improvements in cognitive function may have been observed in those taking antipsychotics.

While specific antipsychotics have a variety of specific receptor interaction profiles, they generally lead to prefrontal cortical activation (57). One common property of second-generation (atypical) antipsychotic is antagonism at serotonin receptors (58). Serotonin antagonism can enhance prefrontal activation either directly (59) or indirectly through dopaminergic mechanisms (60). This process may prime prefrontal cortical regions, leading to a synergistic effect between antipsychotic action and TILS treatment. The most commonly-prescribed antipsychotic in this subject pool, quetiapine, has been shown to increase frontal lobe metabolism during a working memory task (61). Different psychotropic medications are known to have different effects on mitochondrial function (62–64). Quetiapine in particular has been shown to increase brain mitochondrial respiratory chain activity (65).

In addition, the paper-and-pencil trail-making test used here is a psychomotor task, and second-generation antipsychotics can ameliorate negative symptoms of psychosis, such as impaired motor skills (66). Therefore, it is possible that improving mitochondrial function, motor impairment, and prefrontal cortical activation with antipsychotic medication made the TMT-B easier to perform in the medicated participants.

## Limitations

The lack of a sham control group also complicates the interpretation, in terms of assessing how much of the improvement in performance was due to TILS alone vs. practice effects. Considering that the first-week test represents participants’ initial exposure to the 2-back task, it is possible that the results reflect a lack of familiarity rather than a genuine effect. However, we have previously used sham control groups to demonstrate cognitive benefits after TILS (10–13, 67), and the task improvement seen in the current study is consistent with and comparable to these previous findings of cognitive enhancement in neurotypical adults after TILS, so it is likely that at least some of the improvement is due to the TILS treatment. Ultimately, the choice to offer TILS treatment to every participant was based on several reasons, including the extensive time commitment required for the experiment, the difficulty in recruiting this specific population, and an ethical decision to maximize a potential benefit for these individuals. In particular, since significant improvement in cognitive function was observed in participants who continued taking antipsychotics (**Figure 9A**), it is possible that continued antipsychotic medication contributed to the improvement of cognitive function in remitted BD. We considered only the effects of psychotropic medication, but other factors may also be involved. Therefore, to definitely demonstrate TILS efficacy, a sham-controlled, double-blinded randomized trial should be conducted.

Future directions for this line of research include the analysis and publication of additional data gathered as part of this experiment (results from MRI scans and cognitive assessments). We will also explore using graph theory and structural equation modeling to explore the network properties of fNIRS data more fully, as the makers of the NIRSIT device will be incorporating new features into future updates. It is also possible that photosensitizers such as methylene blue may work synergistically with TILS to improve cognition and brain metabolic activity, over and above TILS treatment alone (68). A major limitation of the current work is the lack of a sham-treated control group; including this in the experimental design would allow the disambiguation of practice effects from TILS effects, as well as the assessment of how the observed lateralized fNIRS effects are related to lateralization of function. Future studies should also examine the effects of TILS in participants with BD experiencing depressed or elevated mood states, which could elucidate whether TILS has mood-state-dependent effects on cognitive performance or neuroplasticity (50). Tasks that involve emotional regulation could be particularly useful in these contexts. Finally, the use of light-emitting diodes (LEDs) will be explored as an alternative mechanism for delivering TILS with lasers, as the use of LEDs would be more practical and economical for daily TILS treatments self-administered by people at home (69).

## Conclusion

This work expanded our previous findings of beneficial changes in brain metabolic activity and cognitive enhancement after a single TILS treatment in older adults with BD. Repeated TILS treatments may be effective as a safe, non-invasive, non-pharmacological intervention to improve cognition in people with remitted BD. Further research using randomized sham-controlled trials is supported by the present result and may reveal the potential to ameliorate the cognitive impairment seen in individuals with BD and other psychiatric conditions.

## Data Availability

The raw data supporting the conclusions of this article will be made available by the authors, without undue reservation.
