# Supplementary material for: Cognitive improvement and prefrontal network interactions in individuals with remitted bipolar disorder after transcranial infrared laser stimulation
Source: Front Psychiatry. 2025 Jan 30;16:1547230. doi: 10.3389/fpsyt.2025.1547230 (PMC11822565; doi:10.3389/fpsyt.2025.1547230)
Supplement: Supplementary file 1 [file Table1.docx]

**Supplementary Table 1.** Detailed inclusion/exclusion criteria

| Inclusion criteria: |
| --- |
| 1. Able to read, speak, and understand English. |
| 1. Primary diagnosis of Bipolar Disorder type 1 or 2, by structured clinical interview. |
| 1. Score on the Montgomery-Åsberg Depression Rating Scale (MADRS) ≤ 12. |
| 1. Score on the Young Mania Rating Scale (YMRS) ≤ 7. |
| 1. On a stable and adequate dose of an anti-manic agent and/or mood stabilizer (Lithium with a level of at least 0.6, Depakote with a level of at least 50, or a therapeutic dose of carbamazepine, oxcarbazepine, lamotrigine, or other psychotropic agent for treatment of mania and/or mood stabilization per clinician judgment) without dose changes for at least 6 weeks prior to the active study time period (i.e. first MRI scan). |
| 1. Any standing benzodiazepine to a maximum dose equivalent to 22.5 mg oxazepam or 7.5 mg diazepam per day. |
|  |
| Exclusion criteria: |
| 1. Unable or unwilling to give informed consent. |
| 1. Diagnosed with current primary psychotic disorder (rather than bipolar disorder). |
| 1. Diagnosed with current manic/hypomanic or depressive episode. |
| 1. Moderate to severe substance use disorder within the past 6 months (except nicotine, caffeine, cannabis). |
| 1. Clinically-defined major neurological disorder: including, but not limited to, seizure disorder and history of loss of consciousness due to head injury for greater than 10 minutes, or documented evidence of brain injury. |
| 1. Active suicidal intent/plan as detected on screening assessments, or in the investigator’s opinion likely to attempt suicide within next 6 months. |
| 1. Clinically-significant unstable medical condition. |
| 1. If female: pregnant, not using medically acceptable birth control, or currently breastfeeding. |
| 1. Other condition judged by investigator that could prevent the participant from completion of the study, including significant physical disability (e.g., hearing/visual deficits) impairing ability to perform a neutral memory task and/or neuropsychological test battery. |
| 1. Contraindication for magnetic resonance imaging (MRI) such as ferromagnetic metal implants. |
| 1. Electroconvulsive therapy treatment in the past 6 months. |
| 1. Participants taking clozapine, tricyclic antidepressant, first-generation antipsychotics, and/or benztropine. |
