# Supplementary material for: Cognitive improvement and prefrontal network interactions in individuals with remitted bipolar disorder after transcranial infrared laser stimulation
Source: Front Psychiatry. 2025 Jan 30;16:1547230. doi: 10.3389/fpsyt.2025.1547230 (PMC11822565; doi:10.3389/fpsyt.2025.1547230)
Supplement: Supplementary file 2 [file Table2.docx]

**Supplemental Table 2**. Medications of all participants

| Participant | List of Medications |
| --- | --- |
| 1 | Lithium |
| 2 | Lithium, Lurasidone |
| 3 | Lamotrigine, Amphetamine and Dextroamphetamine |
| 4 | Lamotrigine, Duloxetine, Atomoxetine |
| 5 | Lamotrigine, Escitalopram, Emtricitabine and Tenofovir Alafenamide, Allopurinol |
| 6 | Lithium, Lurasidone |
| 7 | Lithium, Quetiapine, Levothyroxine |
| 8 | Lithium, Fluoxetine |
| 9 | Lamotrigine, Fluoxetine, Lisdexamfetamine |
| 10 | Lamotrigine, Alprazolam |
| 11 | Lithium, Lamotrigine, Quetiapine, Aripiprazole, Paroxetine, Levothyroxine |
| 12 | Lithium, Lamotrigine, Asenapine |
| 13 | Lithium, Quetiapine |
| 14 | Lithium, Lamotrigine |
| 15 | Lithium, Lamotrigine, Sertraline, Atomoxetine |
| 16 | Lithium, Olanzapine, Haloperidol, Gabapentin |
| 17 | Lithium, Quetiapine, Levothyroxine |
| 18 | Lamotrigine, Quetiapine, Lorazepam, Bupropion, Lisdexamfetamine, Gabapentin |
| 19 | Lamotrigine, Amphetamine and Dextroamphetamine, Gabapentin, Zaleplon |
| 20 | Lithium, Quetiapine |
| 21 | Lithium, Lamotrigine, Perphenazine, Levothyroxine, Metformin |
| 22 | Lamotrigine, Propranolol |
| 23 | Lithium, Quetiapine, Bupropion |
| 24 | Lithium, Alprazolam, Gabapentin |
| 25 | Lithium, Quetiapine, Lorazepam, Propranolol |
| 26 | Lamotrigine, Bupropion, Ethinyl estradiol/Etonogestrel vaginal ring |
| 27 | Lamotrigine, Metformin, Dapagliflozin and Metformin, Sitagliptin and Metformin, Alpha Lipoic Acid, Benfotiamine, Chromium Picolinate, Inositol, Methylcobalamin |
| 28 | Lamotrigine, Alprazolam, Duloxetine, Gabapentin, Estradiol, Progesterone, Testosterone, Codeine/Paracetamol, Carisoprodol, Rosuvastatin, Losartan, Hydrochlorothiazide, Hydroxychloroquine, Omeprazole |
| 29 | Lithium, Gabapentin, Spironolactone |
